# Bone health in children and adolescents with perinatal HIV infection

**DOI:** 10.7448/IAS.16.1.18575

**Published:** 2013-06-18

**Authors:** Thanyawee Puthanakit, George K Siberry

**Affiliations:** 1 Department of Pediatrics, Faculty of Medicine, Chulalongkorn University, Bangkok, Thailand; 2 HIVNAT, Thai Red Cross AIDS Research Center, Bangkok, Thailand; 3 Maternal and Pediatric Infectious Disease (MPID) Branch, Eunice Kennedy Shriver National Institutes of Child Health and Human Development, National Institutes of Health, Bethesda, MD, USA

**Keywords:** perinatal HIV infection, bone mineral density (BMD), fracture, dual-energy X-ray absorptiometry (DXA), peak bone mass (PBM)

## Abstract

The long-term impact on bone health of lifelong HIV infection and prolonged ART in growing and developing children is not yet known. Measures of bone health in youth must be interpreted in the context of expected developmental and physiologic changes in bone mass, size, density and strength that occur from fetal through adult life. Low bone mineral density (BMD) appears to be common in perinatally HIV-infected youth, especially outside of high-income settings, but data are limited and interpretation complicated by the need for better pediatric norms. The potential negative effects of tenofovir on BMD and bone mass accrual are of particular concern as this drug may be used more widely in younger children. Emphasizing good nutrition, calcium and vitamin D sufficiency, weight-bearing exercise and avoidance of alcohol and smoking are effective and available approaches to maintain and improve bone health in all settings. More data are needed to inform therapies and monitoring for HIV-infected youth with proven bone fragility. While very limited data suggest lack of marked increase in fracture risk for youth with perinatal HIV infection, the looming concern for these children is that they may fail to attain their expected peak bone mass in early adulthood which could increase their risk for fractures and osteoporosis later in adulthood.

## Introduction

Worldwide, more than 2 million children are infected with HIV. In most of the cases, HIV infection was acquired during pregnancy or intrapartum or through breastfeeding. Effective antiretroviral therapy (ART) has dramatically reduced morbidity and mortality for children with perinatal HIV infection, many of whom are now adolescents or even young adults. Even as the prevention of AIDS-defining illnesses and of progressive immunosuppression is appropriately celebrated, the long-term impact of lifelong HIV infection and prolonged ART in growing and developing children is not yet known. An area of particular concern is the potential effect of HIV infection and ART on bone, which undergoes profound changes in size, mass and strength from foetal life through to young adulthood. This article will focus on available data and remaining questions related to bone outcomes in perinatal HIV infection in the context of normal bone development, non-HIV factors that impact bone, and composition of ART as well as an approach to detection, prevention and management of bone problems in this group.

### Bone assessment definitions and measurement methods

Bone is composed of organic (bone matrix) and mineral components. Bone mass refers to the weight of bone. Bone mineral density (BMD) refers to the bone mass divided by its volume. In practice, BMD is not usually directly ascertainable (would require bone biopsy), and it is estimated by radiologic or other methods. Bone mineralization describes the incorporation of calcium and other minerals into organic bone matrix [1]. Low BMD may result from inadequate bone mass due to inadequate bone matrix, called osteopenia, or from undermineralization of bone matrix, termed osteomalacia [1]. Remodelling of bone is a continual process in which bone is periodically reabsorbed (resorption) and replaced (formation) by new bone; the balance of resorption and formation activity determines whether there is net gain or loss of bone mass. Bone strength is based on bone mass, bone mineralization and bone architecture. Osteoporosis is defined as bone weakness or fragility that manifests as increased susceptibility to fractures and is well correlated with low BMD, especially in older adults.

Dual-energy X-ray absorptiometry (DXA) is the most commonly used modality for estimating bone mineral content (BMC) and BMD. The technique measures how much radiation (two beams emitted at different energy levels) gets absorbed while passing through bone or other body tissue to estimate the density of that region [1]. Denser (calcium-rich) tissues absorb more radiation. The output is expressed as absolute BMC in grams, and, as the ratio of BMC to a projection of three-dimensional bone onto a two-dimensional area to produce the areal BMD (aBMD), usually in grams per square centimetre (g/cm^2^). The main limitation of the aBMD for children is that relatively smaller bones (e.g. in a child with short stature) can lead to lower aBMD and thus underestimates of true BMD [1]. These measures can also be expressed as T-scores, which standardize the absolute results against average results expected at peak bone mass (PBM) for someone of the same sex, and as Z-scores, which standardize the absolute results against average results expected at a population of similar age and sex (and sometimes race/ethnicity). T-scores are primarily used for older adults and are not appropriate for children and young adults; Z-scores can be used at all ages and should be used through to the age when PBM has been achieved [2]. DXA can be used to assess BMD of the total body (with or without head) or of specific body sites; the sites best characterized for DXA assessment in children (lumbar spine and total body) are different from those in adults (lumbar spine and hip) [3]. A DXA BMD T-score<−2.5 in older adults (especially postmenopausal women) has been sufficiently linked to risk of fracture that it can be used as the basis of an osteoporosis diagnosis in that population. The finding of a BMD Z-score<−2.0 in children and youth, however, should be described as “very low BMD for age”; the diagnosis of osteoporosis in paediatrics requires clinical evidence of bone fragility (fracture) [3].

Other modalities used to estimate BMD include quantitative computed tomography (qCT) and quantitative ultrasonography (qUS) (Table 1) [4]. qCT measures bone in three dimensions for a true volumetric BMD and provides information about bone geometry, but it entails relatively high radiation exposure and the technique is not well standardized across centres. qUS measures the attenuation and speed of an ultrasound wave along a bone to estimate BMD; despite the advantages of lack of ionizing radiation and greater portability of ultrasound machines, the lack of standardization of this technique and absence of paediatric reference norms currently limit its use in children. DXA remains the preferred method for bone density assessment in children because of its availability, reproducibility, speed, very low radiation exposure and paediatric reference norms [2,3].

**Table 1:** Common methods for assessing bone mineral density

Modality	Advantages	Disadvantages
DXA	Norms available for children and youth Widely used High reproducibility Short scanning times	Radiation (trivial) Small bone size leads to BMD underestimate Lack of norms for children<age 7 years
qCT	Assessment of three-dimensional (volumetric) bone size and geometry	Radiation Lower reproducibility Whole-body not feasible Lack of norms for children
Ultrasound	No radiation Machines often portable	Lack of norms for children and youth Cannot be used for whole-body or axial skeleton

From refs. [2,4].

DXA=dual-energy X-ray absorptiometry; qCT=quantitative computed tomography; BMD=bone mineral density.

### Normal bone development

**Figure 1:**
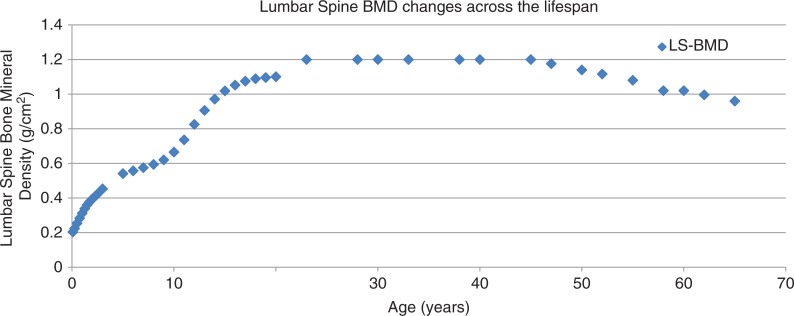
Illustration of changes in lumbar spine bone mineral density (BMD) over the lifespan. Plot based on actual or estimated data from three different studies [5–7].

The potential impact of HIV infection and treatment on the bone health of youth with perinatal HIV infection must be evaluated in the context of normal, physiologic bone growth and development (Table 2). The effect of HIV infection and its treatment on developing bones may well be different from that seen in adults who acquire HIV infection after bone development is complete. Furthermore, the assessment of potential effects must be made relative to normal or expected changes in bone. For instance, BMD, a commonly used measure of bone strength, normally increases throughout childhood and adolescence, peaks and stays relatively constant in healthy adults, and then begins to decline with older age and especially menopause (Figure 1). An assessment of the effect of an antiretroviral drug on BMD (or BMC) must then be considered against the normal age-related expectation for changes in these parameters.

**Table 2:** Normal bone development

Developmental period	Cardinal events	Major factors impacting bone	Other comments
Foetus	- Bone formation - Rapid longitudinal bone growth - Marked bone mineral accretion	- Gestational age - Body size - Most increase in bone mass and growth during third trimester of pregnancy	Entirely dependent on placental transfer of calcium and other minerals
Infant	- Rapid longitudinal bone growth - Marked bone mineral accretion	- Gestational age and body size at birth - Nutrition/breastfeeding, infections, drug and toxin exposures and activity level	Immediate shift to dependence on intestinal absorption, renal reabsorption and bone stores for calcium/minerals
Child	- On-going longitudinal growth and bone mineral accretion (slower pace)	- Nutrition, infections, drug and toxin exposures and activity level - BMI	
Adolescent	- 26% of bone mass in 4-year period of peak height velocity - 60% of adult peak bone mass (PBM) is established	- Puberty - BMI - Age at pubertal onset as well as nutrition, infections, drug and toxin exposures, and activity level - Smoking - Alcohol use - Medroxyprogesterone and other drugs	
Young Adult	- PBM achieved by age 20–25 years (varies by body site)	- BMI - Smoking - Alcohol use - Medroxyprogesterone and other drugs	
Later Adulthood	- No net change in bone mass/density for many years (balanced bone formation and resorption) - Annual declines in BMD after fifth decade, especially with menopause	- Loss of bone with older age - Marked bone loss with menopause - Smoking - Alcohol use - Reduced physical activity - Nutrition	

BMI=body mass index; BMD=bone mineral density; PBM=peak bone mass.

Perinatally infected youth are potentially first exposed to HIV infection and antiretroviral drugs (ARVs) during intrauterine life. The foetal period is a critical period for the development of bone mass. The foetus is entirely dependent upon its mother for calcium and other minerals necessary for normal bone development. Foetal bones accumulate about 30 g of calcium from transplacental transfer, with 80% of calcium accretion taking place in the third trimester [8]. Since calcium deposition into a foetal skeleton increases from 100 mg/day at week 28 to 250 mg/day at week 35 [8], preterm birth substantially compromises the amount of mineral transferred to the foetus and thus has a strong negative effect on newborn bone mineralization status. Maternal malnutrition and calcium deficiency may also contribute to lower foetal bone mineralization, but the effect of these factors appears less certain and less pronounced [9].

Infancy represents an equally critical period in bone growth, development and mineral accretion. In contrast to the foetus, infant bone health depends on maternal (breast milk) and non-maternal sources of calcium and other bone components and is affected by environmental factors, including nutrition, infections, drug and toxin exposures and activity level. During infancy, the increase in bone diameter by 50%, despite a concomitant increase in BMC by at least 50%, results in a decrease in bone density of about 30%. In this complex process, however, actual bone strength increases threefold because of accompanying changes in bone architecture [1]. There are no well-established normative data for BMD (as measured by DXA or other modalities) in infants.

Throughout childhood, bone size, BMC and aBMD continue to increase (Figure 1). Normative DXA BMD and BMC data (based on US children) are available for children aged five years and older [5] which facilitates interpretation of BMD and BMC results in children. There may be advantages to BMC over aBMD DXA measures in children, especially for total body, but both measures are widely used [3]. Healthy children with low aBMD are likely to continue to have low aBMD over time [10]. Some of the biggest and most important changes in bone occur during puberty and adolescence. More than half of lifetime bone calcium is laid down during teen years, and almost half of BMC is accrued in the two years before and after attainment of peak height velocity [11,12]. Delayed puberty may have short- and long-term effects on bone mineral status. Late menarche has been shown to be associated with poorer BMC accrual [13], and the negative effects of late menarche on bone mass may persist up to 25 years [14].

PBM is attained by the end of the second or early in the third decade of life [12], generally occurs earlier at the hip and spine than at the whole body and occurs earlier in girls than in boys. PBM is an important concept for bone health because it has been linked to lifelong BMD outcomes and failing to attain normal PBM by young adulthood increases the risk of bone fragility in later adulthood [15]. In fact, up to 60% of the lifetime risk of osteoporosis may be attributable to the amount of bone mineral accrued through the first two decades of life [16]. Thus, factors that negatively impact bone development in the foetus, child and adolescent may contribute to a compromised PBM in the young adult, but the important clinical consequences may not manifest until decades later as increased risk of fractures and osteoporosis.

### Risk factors for poor bone health (not specific to HIV infection)

Outside the context of HIV infection, there are numerous demographic, genetic, nutritional and lifestyle factors, as well as medical conditions and treatments, that are well known to impact bone health and BMD (Table 3). The relative contribution of these factors to bone health varies by age and setting.

**Table 3:** Non-HIV-specific factors affecting bone health

Factor	Description
Preterm birth	Negative effect increases as gestational age decreases. Short-term fracture risk mainly for very preterm infants.
Abnormal weight (BMI)	Low BMI (general malnutrition and adolescents with eating disorders) associated with low BMD; high BMI (obesity) associated with increased fracture risk.
Specific nutritional deficiency	Inadequate vitamin D and calcium most important. Role of protein and other micronutrients less clear.
Genetic factors	Genetic disorders (osteogenesis imperfecta); family history of osteoporosis; blacks at low risk of osteoporosis relative to other racial/ethnic groups.
Exercise	Weight-bearing activity improves bone mass accrual and BMD; sedentary lifestyle and impaired mobility (as in cerebral palsy) compromise bone health.
Hormones	Normal pubertal increases in endogenous androgens, estrogens and growth hormone promote bone mass accrual. Lower PBM with delayed puberty. Pregnancy and lactation associated with transient BMD decline. Substantial BMD loss and fracture risk with menopause.
Lifestyle factors	Cigarette smoking, alcohol consumption and sedentary lifestyle all impair bone health.
Endocrinopathies	Hypogonadism, hypercortisolism (e.g., Cushing syndrome), hyperthyroidism and growth hormone deficiency associated with poor bone health.
Medications	Well-established negative effect on BMD: corticosteroids, anticonvulsants, medroxyprogesterone. Full list at http://www.nof.org/articles/6.
Inflammation	Juvenile arthritis, inflammatory bowel disease and other inflammatory disorders and conditions; risk related to proinflammatory cytokines and treatment (corticosteroids).
Other medical conditions	Malignancy, renal failure.

BMI=body mass index; BMD=bone mineral density; PBM=peak bone mass.

Intrauterine factors may have both short- and long-term impacts on bone health. Maternal macronutrient and micronutrient intake during pregnancy has been linked to effects on bone mass in progeny through 6–16 years old [17]. Some have hypothesized that this is due to early programming of later bone responses rather than direct consequence of lower bone mass in the foetus [17]. Maternal smoking, likely acting through compromising uteroplacental function, appears to result in lower bone mass in offspring that resolves by the second decade [17].

Preterm birth, low birth weight and poor growth early in life all may have a lasting negative impact on bone health [18,19]. While there is some evidence that breastfeeding, compared to replacement feeding, may result in lower bone mass in infancy, other evidence points to better bone mass and lower fracture risk in older children who were breastfed as infants [17].

Through childhood and adolescence, major influences on bone mass accrual and bone health include genetic determinants, physical activity (or lack thereof), nutritional status and hormonal changes during puberty. Up to 80% of the variability in PBM may be explained by genetic factors [20]. Weight-bearing exercise leads to increased bone mass and BMD; in fact, the level of such exercise during childhood and adolescence may explain almost 20% of PBM attained in early adulthood [21]. Within the normal range of BMI, BMD increases with increasing BMI; however, undernutrition (low BMI, including adolescents with eating disorders) leads to lower BMD and obesity (high BMI) increases the risk of fracture [22,23]. Maintaining adequate vitamin D levels and ensuring adequate calcium intake, especially during adolescence, are associated with better bone outcomes, and insufficiency of either can compromise final PBM [24]. Increases in endogenous androgens, oestrogens and growth hormone accompanying puberty have dramatic effects in promoting increases in bone length, mass and mineral content [23]. Cigarette smoking and drinking alcohol, often beginning in adolescence, both contribute to low BMD and poor bone health [25].

Many illnesses and medications have been associated with low BMD and/or increased fracture risk [23,26]. Osteogenesis imperfecta is a classic example of a genetic disorder associated with extreme bone fragility and high fracture risk. Malignancy and some chemotherapeutic agents used in its treatment increase the risk of fracture in children. Low BMD and fractures are important complications of cerebral palsy and other neurologic disorders associated with reduced mobility. Disorders associated with malabsorption, such as celiac disease and cystic fibrosis, have been linked to poor bone health. Proinflammatory cytokines and glucocorticoid therapy, among other factors, contribute to the elevated risk of fracture and bone structural abnormalities in children with juvenile rheumatoid arthritis and other inflammatory diseases [27]. Hormonal contraception, especially depot medroxyprogesterone, has been linked to significant BMD declines in adolescents most pronounced in the first one to two years of use and largely reversible after discontinuation [28]. Anticonvulsants, methotrexate and many other drugs have been linked to low BMD (http://www.nof.org/articles/6).

### Prevalence of low BMD among HIV-infected children and adolescents

Cross-sectional studies of BMD among HIV-infected youth (Table 4) suggest that the prevalence of low BMD may be lower (<10%) in high-income settings [29,30] than in middle-income settings, such as Thailand (24%) [31] and Brazil (32%) [32]. This difference might be explained by older age, lower nadir CD4 cell, more advanced HIV stage and poorer nutritional status in Thai and Brazil cohorts compared to US and the Netherlands cohorts.

**Table 4:** Prevalence of low bone mineral density among HIV-infected children and adolescents

Reference	Population	Duration of ART (years)	Findings	Associated factors
DiMeglio [29]	*N*=350 Mean age 12.6 years Black 66%, Hispanic 26% and white 8%	9.5 years (IQR 9.1–11.3) 25% had CDC C Nadir CD4 20%	Total body Z-score <−2.0; 7% versus 1% in HIV-negative peers LS Z-score<−2.0; 4% versus 1% in HIV-negative peers	Higher peak viral load and CD4% Ever used indinavir
Bunders [30]	*N=*66 Mean age 6.7 years Black 62%	3.4 years (IQR 1.5–5.2) 72% use PI, mainly nelfinavir	Spinal BMD Z-score<−2.0=8%	
Puthanakit [31]	*N=*100 Age 14.3 years Thai 100%	7.0 years (4.3–8.7) Nadir CD4=114 (31–226) cell/mm3	LS Z-score<−2.0; 24%	Height-for-age Z-score<−1.5 Ever have WHO stage 4
Schtscherbyna [32]	*N*=74 Age 17.3 (SD 1.8) years White 36.5% Non-white 63.5%	11.1 years (SD 3.5) 91% on ART (19% NNRTI, 72% PI)	Low total body or lumbar spine in 32.4% of cohort Use of TDF is associated with lower lumbar spine Z-score: −1.8 (1.1) vs. −1.3 (0.9) Use of protease inhibitor is associated with LS Z-score −1.7 (1.1) vs. −1.1 (0.9)	Weight, BMI, nutrition, use of tenofovir and protease inhibitors

ART=antiretroviral therapy; N=number; IQR=interquartile ratio; PI=protease inhibitor; NNRTI=non-nucleoside reverse transcriptase inhibitor; BMD=bone mineral density; LS=lumbar spine; SD=standard deviation; BMI=body mass index.

In one longitudinal study, BMD increased over one year in 32 HIV-infected Italian children with a mean age of 12.4 years, but it remained significantly lower than HIV-uninfected controls [33].

Many [34–37] but not all [38] studies have demonstrated an increased fracture risk in adults with HIV infection. There is no clear evidence of increased fracture risk in HIV-infected children in the United States [39] or Latin America [40], but these negative studies are not definitive.

Studies of BMD and fracture risk in HIV-infected youth in low-income settings have not been published.

### Factors affecting BMD among HIV-infected children and adolescents

Many factors contribute to low PBM in children with HIV infection, including delayed growth and puberty, low lean body mass, altered levels of hormones and inflammatory cytokines, vitamin D deficiency, malabsorption and physical inactivity. HIV-specific factors, which are important contributing factors to bone loss in children, include advanced HIV disease, uncontrolled viremia and ART initiation and type [41]. A large study of 236 HIV-infected US children aged 7–24 years showed that HIV-infected males had significantly lower BMD at Tanner stage 5 compared to HIV-uninfected males [42]. Among HIV-infected Thai adolescents, WHO stage 4 increased by 3.4 times the risk of low BMD, and height-for-age Z-score<−1.5 made low BMD 6.2 times more likely [31]. In adult studies, the period after ART initiation is associated with BMD loss [43,44], and lower cumulative ART use through structured treatment interruptions is associated with better BMD outcomes [45], suggesting a negative BMD effect of ART in general. Studies of BMD effect of ART initiation in treatment-naïve children are not available but longer ART duration in one longitudinal study of 66 Dutch children was reassuringly associated with increases in spinal BMD Z-scores [30].

Not all antiretroviral drugs have the same effect on BMD. Protease inhibitors and tenofovir are most often associated with low BMD. Lopinavir/ritonavir [42], indinavir [29] and full-dose ritonavir [46] were associated with lower BMD in children. In trials randomizing treatment-naïve adults to tenofovir- vs. abacavir-containing ART, BMD decreased in both arms, but BMD loss was significantly greater in the tenofovir arm [43,44]. Tenofovir was also associated with a yearly hazard ratio for osteoporotic fracture of 1.12 (95% CI 1.03–1.21) in HIV-infected adults [47]. Tenofovir was recently approved for children as young as two years but there is concern about potentially greater effects of tenofovir on developing bones in these young children. In a US study, median Z-scores of BMD of the lumbar spine, femoral neck, and total hip decreased from baseline at weeks 24 and 48, and remained stable up to week 96. The children who experienced >1% decrease in BMD were significantly younger than those with stable BMD (10.2 vs. 13.2 years, *p*=0.003) [48]. On the other hand, some studies reported no effect of tenofovir on BMD. In 16 Italian children (6–18 years) receiving suppressive ART regimens, replacing stavudine and PI with tenofovir and efavirenz did not result in smaller 12-month BMC or BMD increases relative to HIV-uninfected peers [49]. Another study of 21 Italian children receiving tenofovir/efavirenz/lamivudine documented no significant change in BMD Z-score from baseline through 60 months [50]. The lack of a negative effect on BMD observed in these studies may be explained by the use of a lower dose of tenofovir, lack of concomitant PIs and ART switch (instead of ART initiation). In a placebo-controlled US/Panama/Brazil trial of tenofovir-containing ART for 87 youth (12–17 years) with virologic failure of their current ART regimen, there was no significant difference in 48-week BMD between the tenofovir and non-tenofovir arms, but there was a trend for more tenofovir-arm than non-tenofovir-arm subjects to have spine BMD losses >4% (18% vs. 3%, *p*=0.1) [51]. There are no published studies of initiating tenofovir-containing ART in treatment-naïve children. Due to limited information regarding long-term effects on bone development in young children, the US DHHS guidelines recommend tenofovir (as part of initial ART) for adolescents in Tanner stages 4–5, as an alternative for those in Tanner stage 3, and only for special circumstances for children in Tanner stages 1–2 [52].

### Pathogenesis of low BMD among HIV-infected individuals

The pathogenesis of low bone mass among HIV-infected individuals is multifactorial, including traditional risk factors such as smoking, physical inactivity and vitamin D deficiency and also HIV-related factors including HIV infection itself, chronic immune activation and the direct effects of antiretroviral therapy. In vitro studies have shown that HIV viral proteins gp120 [53] and Vpr [54] stimulate osteoclast activity, and p55-gag suppresses osteoblast activity and increases osteoblast aopotosis [55]. Osteoclasts (OCs), the cells responsible for bone resorption, form from precursors that circulate within the monocytic population, and are recognized by their expression of receptor activator of NF-κB (RANK). OC precursors differentiate into OCs under the influence of the key osteoclastogenic cytokine, RANK ligand (RANKL), moderated by RANKL's physiological decoy receptor osteoprotegerin (OPG) [56]. In HIV-1 transgenic rat model, there is a significant increase in total RANKL expression concomitant with a significant decline in total OPG expression in both bone marrow and spleen [57]. HIV-infected antiretroviral naïve adults with low BMD had elevated RANKL/OPG ratio [58]. Similarly, perinatally HIV-infected children had elevated RANKL/OPG ratio compared with healthy children [59]. Activation of T-cells by HIV infection may also affect bone physiology by producing RANKL and pro-inflammatory cytokines (e.g., IL-1 and TNF-α), which promote osteoclast activity and stimulate stromal cells to produce osteoclastogenic IL-7. Finally, CD4+ and CD8+ T-cell activation has been independently associated with low bone mineral study [60]. Further research is needed to fully characterize the pathogenic processes leading to low bone mass in the context of HIV infection.

### Approach to the assessment of bone health in HIV-infected children and adolescents

Careful review of occurrence and circumstances of fracture can help identify children and youth with increased bone fragility. Fractures that occur after minimal trauma, vertebral compression fractures, a single instance of traumatic fracture of a lower extremity long bone, and two or more fractures of upper extremity long bones should raise suspicion of bone fragility [61]. In lower resource settings where DXA scanning is not available, fracture history and review of risk factors for poor bone health (as discussed above) may be the only means to assess potential bone fragility in HIV-infected children. In less resource-constrained settings, BMD assessment by DXA provides important information about risk of skeletal fragility.

There is no consensus that all HIV-infected youth (or adults) should undergo routine DXA screening. If DXA is available, it should be considered for HIV-infected youth with a suspicious fracture history and/or multiple risk factors for poor bone health. As for children in general, lumbar spine and total body less head are the sites recommended for assessments. The hip is not a reliable site for measurement in growing children due to significant variability in skeletal development and lack of reproducibility [3]. A BMD Z-score less than −2, categorized as low BMD, can be used to corroborate a suspicious fracture history for a diagnosis of osteoporosis; in the absence of fractures, it identifies children who are at increased risk of bone fragility and fractures.

The normative databases used as a reference should be based on large samples of healthy children that are similar in gender, age and race/ethnicity [3]. For example, in the study among Thai HIV-infected children, 24% of children had a BMD Z-score ≤−2.0 using Thai children normative data; this prevalence would have been 43% by the Caucasian normative data generated by the DXA scanner database [31]. Therefore, it is very difficult to interpret BMD measurements among HIV-infected children in settings where the normative data for specific ethnicity is not available. Furthermore, the DXA interpretation must be specific for each manufacturer and model of densitometer and software. There are three dominant DXA manufacturers: Hologic Inc. (Bedford, MA, USA), GE-Lunar Inc. (Madison, WI, USA) and Copper Surgical (Norland; Trumbull, CT, USA). They are different in calibration standards, proprietary algorithms to calculate the BMD and in the regions of interest (ROI). As a result, a patient scanned on different DXA systems will have substantially different BMD values, e.g. Hologic spine BMD is typically 11.7% lower than GE-Lunar BMD and 0.6% higher than Norland BMD [62].

Delay in growth and puberty is quite common among HIV-infected children. BMD interpretation should be adjusted for absolute height or height age in children with linear growth or maturational delay [3]. Substituting height age for chronological age as a means of adjusting for short stature may not be a preferred approach because it may treat as similar children with the same height who are at different stages of sexual maturation. Using height-for-age Z-score adjustment may result in the least bias, but the equation for height-for-age Z-score adjustment is developed only for healthy US children based on the Hologic system [63]. Adjusting for bone age, pubertal stage or lean mass has also been studied.

In children who have a baseline BMD assessment by DXA, there is no clear recommendation for how often DXA scans should be repeated as part of monitoring bone health. Intervals shorter than six months are unlikely to yield significant changes in BMD; intervals of one to two years may be reasonable for children with low BMD at baseline or ongoing risk for skeletal losses [3].

### Prevention strategies for addressing bone health in children/adolescents with HIV infection (in high and lower income settings)

Many of the approaches to maintaining good bone health in youth with perinatal HIV infection are similar to those used for youth in general (Table 5). Adolescents should receive at least 1300 mg calcium/day and at least 600 IU vitamin D/day through their diet and/or supplementation [64]. Vitamin D deficiency is very common in youth with or without HIV infection [66]. In addition, there is evidence for interference with normal vitamin D metabolism by some ARV medications [41] and for elevation in PTH in youth who take tenofovir [67], which may mean that HIV-infected youth need higher doses of vitamin D to achieve functional vitamin D sufficiency. If the measurement of blood levels of 25-OH vitamin D is available, supplementation can be initiated and adjusted based on these measured levels. However, in many settings where 25-OH vitamin D measurement may be costly and impractical, clinicians should provide vitamin D supplementation if intake by history seems insufficient. General guidance about good nutrition and counselling to avoid or stop cigarette smoking and alcohol consumption should be routine. Regular, weight-bearing exercise should be promoted. Such exercise does not require sophisticated equipment or facilities; running, jumping or playing a sport like basketball are effective and feasible options for most youth. Note that swimming does not involve weight-bearing or impact and so would not have any benefit to bone health. Minimize the use of corticosteroids and other medications with negative impact on bone health, but only after assessing relative risks and benefits. For example, the benefits of medroxyprogesterone for effective contraception in youth likely outweigh the negative effects on BMD [28].

**Table 5:** Prevention strategies to optimize bone health in perinatally HIV-infected youth

Calcium+vitamin D	Ensure adequate intake of calcium (1300 mg/day) and vitamin D (600 IU/day) in adolescents [64].
Promote healthy lifestyle	Good nutrition; avoid/stop cigarette smoking; avoid/limit alcohol consumption.
Exercise	Encourage high-intensity impact activities (like running, jumping, gymnastics, basketball) for 10–20 min/day at least three days/week [65].
Effective ART	Regardless of the specific regimen, ART that achieves virologic suppression, preserves/restores immunologic function, and minimizes HIV-related illnesses should have a generally positive effect on bone health.
Avoid bone “unfriendly” medications	Individualized risk-benefit assessment critical. Minimize use of systemic corticosteroids. For youth with multiple risk factors for poor bone health, consider avoiding TDF, boosted PIs, medroxyprogesterone.

ART=antiretroviral therapy; TDF=tenofovir disoproxil fumarate; PI=protease inhibitor.

Even though continuous ART resulted in lower BMD than intermittent BMD in adults in the SMART study [45], untreated HIV infection that results in progressive immunodeficiency, weight loss and opportunistic illnesses is likely to have a more negative effect on bone health [31,68]. For youth who have multiple other risk factors for low BMD, consideration of regimens that do not include TDF and/or boosted PIs is reasonable but only if an alternative ARV regimen is expected to achieve virologic suppression and be well tolerated.

### Intervention/treatment strategies for HIV-infected youth with evidence of bone fragility (in high and lower income settings)

For youth with perinatal HIV infection who have very low BMD (Z-score<−2.0) and/or fractures that are suspicious for bone fragility, clinicians should implement a multi-pronged approach (Table 6). Calcium, vitamin D and general nutritional sufficiency must be assured and may be facilitated by involving a nutritionist. Consider routine supplementation with calcium and vitamin D. If 25-OH vitamin D is measured, a reasonable target is 30 ng/dL though consensus on this target is lacking. There are several approaches to treating vitamin D deficiency in youth [69]. Weight-bearing exercise should be emphasized; consider collaboration with a physical therapist or other allied health professional to enhance adherence to the exercise regimen. Avoidance of cigarette smoking and alcohol use must be stressed. The risk-benefit of the use of medications like medroxyprogresterone should be reassessed and alternatives should be considered.

**Table 6:** Intervention strategies for perinatally HIV-infected youth with evidence of bone fragility (low BMD, fractures)

Calcium+vitamin D	Provide routine calcium (1300 mg/day) and vitamin D (600 IU/day) supplementation for youth, unless intake history for calcium and measured 25-OH vitamin D levels, respectively, confirm sufficiency. No consensus on target 25-OH vitamin D level, but consider higher threshold (≥30 ng/dL) +/− normal PTH level in youth with bone fragility.
General nutrition	Consider referral to nutritionist for in-depth counselling.
Modify habits	Emphasize importance of not smoking and avoiding alcohol consumption.
Weight-bearing exercise	Prescribe high-intensity impact activities (like running, jumping, gymnastics, basketball) for 10–20 min/day at least three days/ week. Consider referral to physical therapist to improve adherence to exercise regimen.
Reexamine need, or potential substitutes, for non-HIV medications	Avoid or minimize corticosteroids. Consider switching from medroxyprogesterone to alternative contraception. Review list of other agents with potential negative impact on BMD: http://www.nof.org/articles/6.
HIV virologic suppression	Review regimen and optimize adherence to ensure sustained effective ART.
Bone-friendlier ARV regimen	Consider replacing TDF (and/or boosted PI) with other ARV(s), if new regimen anticipated to maintain virologic suppression and be well tolerated.
Anti-resorptives: bisphosphonates	Proven effective (alendronate) in improving BMD in HIV-infected adults and in non-HIV-infected youth with bone fragility. Investigational in youth with HIV infection. Recommend consultation with endocrinologist or other bone specialist.
Other osteoporosis agents	No data for use of other osteoporosis agents (e.g. Denosumab, Teriparatide, Strontium, Raloxifene).

BMD=bone mineral density; ARV=antiretroviral drug; ART=antiretroviral therapy; TDF=tenofovir disoproxil fumarate; PI=protease inhibitor.

Alternative ARV agents can be considered. In particular, replacing tenofovir and/or boosted PIs with agents that have not been associated with negative bone effects (e.g. ABC for TDF; NNRTI or integrase inhibitors for boosted PI) may be beneficial but evidence is lacking. Such regimen changes should only be undertaken if the new regimen is expected to be sustainable, maintain virologic suppression and be well tolerated.

Drugs like bisphosphonates that inhibit bone resorption have been widely used to treat osteoporosis, especially in post-menopausal women. Alendronate, one of the most widely used oral bisphosphonates, improved BMD in HIV-infected adults with low BMD in a randomized, placebo-controlled trial [70]. This drug has also been used in children with other causes of osteoporosis but there is uncertainty about the long-term impact of anti-resorptive drugs on growing bone [71]. Trials in HIV-infected youth have not been completed. Alendronate, along with the measures described above, may be useful for the management of perinatal HIV-infected youth with persistent bone fragility, but this treatment should be undertaken in consultation with an endocrinologist or other bone specialists. There are no data to support newer classes of osteoporosis agents in children and youth.

## Conclusions

Measures of bone health in youth must be interpreted in the context of expected developmental and physiologic changes in bone mass, size, density and strength that occur from foetal through adult life. The potential effects of HIV infection, ARV drugs and other factors on the bones of perinatally HIV-infected youth begin in utero and persist through the critical bone growth and development periods during childhood and adolescence and into young adulthood when PBM is attained. Low BMD appears to be more common in perinatally HIV-infected youth in lower resource settings, likely due to differences in genetic/ethnic and environmental factors, but data are limited and are complicated by the lack of well-characterized paediatric DXA BMD norms for each setting. The potential negative effects of tenofovir on BMD and bone mass accrual are of particular concern as this drug may be used more widely in younger children. Emphasizing good nutrition, calcium and vitamin D sufficiency, weight-bearing exercise and avoidance of drugs (medications in addition to cigarettes and alcohol) are effective and available approaches to maintain and improve bone health in all settings. More data are needed to inform therapies and monitoring for HIV-infected youth with proven bone fragility. While very limited data (with no data from low-resource settings) suggest lack of a marked increase in fracture risk for youth with perinatal HIV infection, the looming concern for these children is that they may fail to attain their expected PBM in early adulthood which could increase their risk for fractures and osteoporosis later in adulthood.
